# Electronic Logistic Management Information System in Public Health Facilities and Its Implications for the Medicine Supply Chain in Singida District Council, Tanzania

**DOI:** 10.3390/pharmacy12040112

**Published:** 2024-07-18

**Authors:** Anwar Milulu, Stanley Mwita, Namanya Basinda

**Affiliations:** 1 Department of Community Medicine, School of Public Health, Catholic University of Health and Allied Sciences, Mwanza 33102, Tanzania; 2 Department of Pharmaceutics and Pharmacy Practice, School of Pharmacy, Catholic University of Health and Allied Sciences, Mwanza 33102, Tanzania

**Keywords:** electronic logistics management information system, health facilities, medicine supply chain, Tanzania

## Abstract

The effective management of the medicine supply chain is crucial for ensuring the availability of essential medicines and supplies in public health facilities. This study aimed to determine the utilization of the electronic logistic management information system (e-LMIS) in public health facilities and its implications for the medicine supply chain. A mixed methods approach, combining both quantitative and qualitative data collection methods, was used. The study included 106 healthcare providers from 35 public health facilities in Singida District. Six key informants were interviewed using a qualitative method. Of the 106 participants, 62.3% said they were somehow competent in e-LMIS utilization. In in-depth interviews, respondents underscored the system’s utility for tracking stock levels, procurements, and managing orders. Staff shortages and a lack of customized training were mentioned as major challenges hindering efficiency in managing drug supplies. This study highlighted the positive impact of e-LMIS on various aspects of the medicine supply chain, including the timely submission of orders and enhanced inventory management. Sustained management support and the regular utilization of the e-LMIS system are crucial for building and maintaining competence among healthcare providers, thereby optimizing the medicine supply chain and ultimately improving healthcare delivery.

## 1. Background

The effective management of the medicine supply chain is crucial for ensuring the availability of essential medicines and supplies in public health facilities [1]. The logistics management information system ensures commodity availability, facilitates service seeking in the community, and enhances the quality of care [2]. Competence in the Electronic Logistic Management Information System (e-LMIS) is essential for supporting the supply chain by enabling effective system utilization, encompassing skills such as data management, procurement, and distribution of medicines [3]. In the supply chain cycle of health commodities, procurement, distribution, and inventory management are crucial stages that can significantly impact the products’ accessibility and affordability for end-users [4].

The main drivers associated with an effective supply chain are demand management, institutional framework, good governance, and management involvement in decision-making regarding e-LMIS implementation [5]. The functionality of health facility management teams is crucial for overseeing daily operations and medicine supplies, especially in the context of quality healthcare service delivery [6]. However, the majority of healthcare workers in public health facilities in developing countries often lack competence in information systems due to barriers such as lack of information technology training, workload, and the availability of the Internet, computers, and electricity [7]. The Tanzanian Ministry of Health has organized the flow of health commodities and their information according to various levels, from the dispensary level to the Medical Store Department (MSD) central office [8]. To improve the supply chain of health commodities and forecasting that relies on reliable and accurate data and information at various levels, the Tanzanian Ministry of Health introduced and implemented an e-LMIS in all districts [9]. Despite the introduction of the e-LMIS in Tanzania, the medical commodities’ supply chain is still a challenge [5,10].

User competence is paramount for the successful implementation of e-LMIS [11]. Given the fact that e-LMIS is implemented in the context of low-resource settings, there is a need to evaluate its impact on medicine supply chain performance. However, there is a scarcity of research that has been conducted to assess the competence of healthcare workers at public health facilities. Thus, this study aimed to determine the utilization of e-LMIS in public health facilities and its implications for the medicine supply chain in Singida District Council, Tanzania. By addressing this research gap, evidence-based solutions can be developed to improve e-LMIS management and enhance the availability of essential medicines in public health facilities.

## 2. Methodology

### 2.1. Study Area

The study was conducted in Singida District Council, part of Tanzania’s Singida Region. The district covers an area of 3787 square kilometers and serves a population of about 304,616 as of the 2022 population census [12]. Within the district are 39 health facilities, including hospitals, health centers, and dispensaries. 

### 2.2. Study Design and Study Population

This study employed a mixed methods approach, combining both quantitative and qualitative data collection methods. The study was conducted from July to September 2023. Quantitatively, it utilized a descriptive cross-sectional study design through surveys. Qualitatively, key informant interviews were conducted. A total of 106 healthcare workers were selected as the study’s sample size based on the Taro Yamane Formula for population sampling. A total of 35 primary health facilities were purposefully selected from 39 health facilities present in the district. Then, healthcare workers dealing with e-LMIS activities were conveniently recruited from each facility to be involved in the study. This study included healthcare workers, i.e., nurses, medical officers, pharmaceutical personnel, and staff involved in the medicine supply chain management process in public health facilities in Singida District. They were selected based on their willingness to participate and their availability during data collection. Six key informants were also selected purposefully for in-depth interviews. Healthcare providers who did not receive training on e-LMIS, those who were not involved in e-LMIS activities on a daily basis, and those who were not at work during the day of data collection were excluded.

### 2.3. Data Collection Method

#### 2.3.1. Quantitative Data Collection

The quantitative data for this study were collected through a self-administered structured questionnaire and a competence assessment tool. A team of three trained research assistants was recruited to ensure the effective collection of data. Two experts from the School of Public Health reviewed and validated the content of the questionnaire to ensure that the questionnaire items adequately covered the content domain that the study aimed to measure. The panel selected the best items for clarity and accuracy in the questions. Additionally, this panel assisted in determining and evaluating the items that were first chosen for inclusion in the questionnaire in terms of their content validity (relevance, coverage, and representativeness). A tool used to assess the level of competence in e-LMIS consisted of 13 items that gathered information on information processing, content creation, communication, problem solving, and the safety of the system. Each item was scored as very competent, competent, somehow competent, or not competent as per the respective number of responses. The average score per scale as per participant responses was used to calculate the overall competence level.

#### 2.3.2. Qualitative Data Collection

The qualitative data for this study were collected through in-depth interviews with key informants. During these interviews, digital recorders were used to ensure that no valuable information was missed. Prior permission to use a voice recorder was obtained from the participants. 

Six key informants were purposefully selected for in-depth interviews. Two in-charges were randomly selected from two dispensaries, two key informants from two health centers, and the remaining two participants from the district hospital. These key informants provided valuable insights into the factors influencing the competence of healthcare workers in utilizing e-LMIS and its impact on the management of the medicine supply chain in health facilities in Singida District.

### 2.4. Data Management and Analysis

#### 2.4.1. Quantitative Data Management and Analysis

Data were coded, entered, cleaned, and analyzed using the Statistical Package for Social Studies (SPSS) version 26. Demographic characteristics, e-LMIS utilization, and competence were analyzed using descriptive statistics. Data were presented as frequencies and percentages. 

#### 2.4.2. Qualitative Data Management and Analysis

Qualitative data obtained from in-depth interviews underwent content analysis, following a structured process. Initially, audio files were transcribed verbatim and translated into English. The data were organized by categorizing and familiarizing information from the interview transcripts and grouping responses under each relevant topic and unique question. Repeated concepts, patterns, and ideas were then identified, with repeated words or ideas being observed and classified. Inductive coding was employed, and codes were assigned to categories. This coding process was carried out separately by two individuals to minimize intercoder variability, and codes with a high degree of agreement were retained. Subsequently, data concepts were synthesized into overarching themes to provide an in-depth understanding of the data. These resulting themes were then described, and the relationships between them were interpreted. To facilitate this qualitative data analysis process, the NVivo 11 software was utilized.

### 2.5. Ethical Consideration

The study obtained ethical approval from the Joint Catholic University of Health and Allied Sciences and Bugando Medical Centre Research and Ethics Review Committee, with permit number CREC/691/2023, approved on 14 July 2023. Permissions were also obtained from relevant authorities, and informed consent was sought from the study participants. Confidentiality and privacy were strictly maintained during the data collection process.

## 3. Results 

### 3.1. Results from the Quantitative Method

#### 3.1.1. User Perceptions of e-LMIS Utilization

About 42.5% reported being very confident in fulfilling e-LMIS duties. All participants had access to computers. Furthermore, most respondents (80.2%) reported that e-LMIS is user-friendly and easy to use. The majority of participants (62.3%) reported placing orders once per quarter. Regarding the timeliness of request revisions (R&R) sent to council supervisors, 67.9% submitted their orders within the stipulated time (Table 1).

#### 3.1.2. Competence Levels of Study Participants on e-LMIS

Most respondents (62.3%) said they were somehow competent in e-LMIS utilization. Almost one-third of study participants reported being competent in e-LMIS utilization. The following parameters were reported as competent by more than one-third of participants: Ability to search, find, and retrieve information using e-LMIS (35.8%); Evaluation of information obtained from e-LMIS (37.7%); Adjusting settings and customizing features in e-LMIS based on needs (35.8%); Familiarity with data visualization tools and techniques (35.8%); Sharing and collaborating on information using e-LMIS (35.8%); and Interacting with colleagues and stakeholders through e-LMIS (35.8%) (Table 2).

### 3.2. Results from the In-Depth Interviews

In this study, six participants were interviewed to gain insights into their experiences with the e-LMIS and its impact on drug availability within their healthcare facilities. 

#### 3.2.1. Theme 1: Competence with e-LMIS

The participants’ interactions with the e-LMIS reveal insights into their daily use and specific applications. Participant 6 emphasized their daily reliance on the system, particularly for checking drug availability.

Participant 6: “I use the system daily, mainly for checking drug availability during this period.”

Participant 3 emphasized the system’s utility for tracking stock levels and managing orders. This specific use case underscores the e-LMIS’s pivotal role in maintaining an accurate inventory and streamlining the procurement process. It enables users to stay informed about stock levels, ensuring timely replenishment and preventing stockouts.

Participant 3: “I use it mainly for tracking stock levels and managing orders.”

In addition to daily use, the participants employ the e-LMIS for specific use cases, such as tracking stock levels, managing orders, and identifying overstocked items. Participant 2’s description of their role in medical supply procurement demonstrates this specificity. They clarified how requests are submitted and approved through the system, highlighting its essential role in their responsibilities.

Participant 2: “In my role, when the relevant department needs to procure medical supplies, they identify what is required and submit the requests to administration. We then review the availability of funds to approve the orders for necessary items.”

Despite varying competence levels, the participants candidly expressed the challenges stemming from their competence levels when engaging with the e-LMIS. Participant 1 underscored the limiting impact of inadequate specialized training on their effective utilization of the system, emphasizing the significance of tailored instruction.

Participant 1: “The lack of specialized training limits my ability to fully utilize the system effectively.”

Participant 4 identified staff shortage as a major challenge, hindering efficiency in managing drug supplies.

Participant 4: “The main challenge is the shortage of staff. We have a heavy workload, and sometimes it’s difficult to find someone who can assist when needed, which can affect efficiency.”

Furthermore, participants exhibited areas of strength and expertise within their utilization of the e-LMIS. Participant 3 excelled in tracking drug availability during specific periods, signifying a valuable skill for ensuring timely access to essential medications.

Participant 3: “I would say I feel most proficient in managing the general drug orders.”

Participant 5 demonstrated competence in identifying both drug availability and overstocked items, underscoring their effectiveness in optimizing inventory levels.

Participant 5: “In finding drug availability and identifying overstocked items, I feel proficient.”

#### 3.2.2. Theme 2: Factors Influencing Competence

The participants cited several factors contributing to their competence in using the e-LMIS. Among these factors, the regular usage of the system was highlighted as instrumental in building competence. Competence in navigating the complexities of the e-LMIS is a dynamic process that the participants attributed to continuous skill development. As Participant 5 emphasized, competence emerges through consistent engagement with the system, marked by regular data entry and the vigilant monitoring of available items.

Participant 5: “Competence comes from using it regularly, entering data, and checking the available items in the system.”

Training and support, both from experienced colleagues and the Ministry of Health, played pivotal roles in refining their abilities. Participant 2 underscored the importance of cooperative relationships with colleagues who possessed both training and practical experience in using the system. This collaborative learning environment contributed significantly to the participants’ capacity to effectively navigate the e-LMIS.

Participant 2: “The main contributing factor has been the cooperation and support I receive from my colleagues who have both training and experience in using the system. Training provided by fellow pharmacists and the Ministry of Health has been valuable.”

Additionally, the presence of educational resources played a pivotal role in supporting the informants’ competence levels. The availability of training sessions and guidebooks was identified as beneficial.

Participant 1: “We received training, and we also have guidebooks that help. These resources serve as valuable references for troubleshooting and skill development.”

Furthermore, management support was acknowledged as a significant influence on competence. The role of management in encouraging the utilization of the e-LMIS was noted to be pivotal in boosting competence. This support was seen as a catalyst for increasing the adoption and effective use of the e-LMIS.

Participant 5: “Management support helps improve our services,” underscoring the pivotal role of leadership in this context.”

Access to experienced pharmacists, especially for consultation when facing system-related issues, proved invaluable. The reliance on consultation with the regional pharmacist exemplified how such support mechanisms aided participants in resolving challenges and optimizing their utilization of the e-LMIS.

Participant 4: “I consult with the regional pharmacist if I have any issues or concerns regarding the system. Consulting with experienced pharmacists when facing issues or concerns is essential.”

#### 3.2.3. Theme 3: e-LMIS Implications on the Medicine Supply Chain

Competence in utilizing the e-LMIS yields a range of positive outcomes that significantly influence the efficiency of the drug supply chain. The respondents noted that orders were received promptly, and the drugs requested aligned with what was delivered. This marked an improvement from the past when discrepancies were common.

Participant 6: “We now receive drugs on time, and the drugs we order are the ones we receive.” These positive outcomes underscore the significance of competence in e-LMIS utilization. The participants described how their competence played a vital role in preventing stockouts and enhancing decision-making processes. The real-time insight not only prevents stockouts but also streamlines resource allocation by ensuring that orders align with the actual requirements rather than relying on manual records and guesswork.

Participant 1: “e-LMIS has positively affected decision-making because I can access real-time information. This allows me to make informed decisions based on the current situation, which is more efficient than relying on manual records. We have not experienced stockouts for a long time because we receive accurate information.”

Furthermore, competence directly influences the decision-making processes of the participants, enabling them to make strategic choices regarding which items to order.

Participant 5: “e-LMIS influences my decisions in that I know what to do next time, like whether to order a certain item or not.”

Participant 1: “e-LMIS has positively affected decision-making because I can access real-time information.”

#### 3.2.4. Theme 4: Strategies for Improvement

The participants stressed the significance of regular training programs and capacity-building initiatives. They advocated for the provision of consistent and structured training to strengthen skills in navigating the e-LMIS effectively. Such training programs, tailored to the needs of healthcare workers, can play a pivotal role in keeping participants updated on system advancements, best practices, and emerging trends.

Participant 6: “I think regular training should be provided to strengthen skills in how to use the system. I believe they should refresh us if there are any updates.”

Participant 1: “Dedicated training programs would be extremely beneficial.”

Additionally, the participants offered insightful recommendations for system enhancements. One prominent suggestion was the automation of certain items’ inclusion in the e-LMIS. The automation would not only streamline processes but also mitigate the risk of errors, as items vital to healthcare operations would already be integrated into the system. Such enhancements can simplify the workflow for healthcare workers, reduce the burden of data entry, and further enhance the overall efficiency of the e-LMIS.

Participant 5: “It would be good if, for example, certain items that should be in the hospitals were automatically in the system.”

Moreover, informants proposed simplifying the e-LMIS itself, making it more user-friendly. Given its extensive functionalities, simplification was seen as a means to enhance user experience.

Participant 3: “Another way is to improve the system itself. It’s quite extensive, so if there’s a way to simplify it, that would be helpful.” This suggestion aligns with making the system more accessible and efficient.

## 4. Discussion

The effectiveness of the various procedures and aspects of the supply chain system, such as storage, transportation, planning, and forecasting, depends greatly on the logistics management information system [13]. This study assessed the competence of the e-LMIS among public healthcare workers and its implications for medicine supply chain performance. The majority of study participants in the present study reported being at least somehow competent in utilization and confident in their supply chain duties using the e-LMIS. This is consistent with a previous study conducted in Ethiopia, where about 95% of respondents reported being satisfied with their ability to use the e-LIMS for inventory management and managing day-to-day activities [14]. More than three-quarters of the study participants reported being either confident or very confident in their ability to fulfill e-LMIS duties. This indicates a significant level of confidence, which is a positive sign of the system’s acceptance and reliability. All participants had access to computers. This universal access is a vital requirement for the effective implementation and use of the e-LMIS. A majority (80.2%) found the e-LMIS user-friendly and easy to use. High usability is crucial for the adoption and sustained use of any new system, suggesting that the e-LMIS is well-designed and user-friendly [14]. The majority (62.3%) reported placing orders once per quarter. However, the timely reporting rate in this study is low compared to the findings from the Ethiopian study (69.4%) [2]. Quarterly order placement aligns with standard supply chain practices in many public health systems, indicating that users are following the expected protocols. About two-thirds of participants submitted their orders within the stipulated time. Timely submission is essential for maintaining an efficient supply chain and avoiding stockouts or overstocking of medicines [15]. More than one-third of study participants reported being competent in the ability to search for, find, and retrieve information; evaluate information; adjust settings and customize features; be familiar with data visualization tools; share and collaborate on information; and interact with colleagues and stakeholders. Although the competence levels reported in this study are promising, there is room for improvement. Further training and capacity building are necessary to enhance these skills across all users. Specific training programs could focus on improving skills in using digital devices and networks to communicate with other stakeholders, seeking digital solutions when encountering difficulties in the e-LMIS, manipulating data and generating reports in the e-LMIS, and sorting and organizing information within the e-LMIS.

The in-depth interviews highlighted several factors that influence competence in the utilization of the e-LIMS. The participants cited several aspects contributing to their competence in using the e-LMIS. These factors were the regular usage of the system; training, and support, both from experienced colleagues and the Ministry of Health; and the presence of educational resources. Other factors were management support and access to experienced pharmacists for consultation when facing system-related issues. Previous studies reported training, educational status, the availability of automated record systems, supportive supervision, and years of work experience in supply chain management as factors associated with competence in the utilization of the e-LIMS [2,16].

The e-LMIS should provide timely reports that help decision-makers and managers to make logistical decisions and manage the supply chain. The system was designed to enhance stock availability, which can be attributed to the system’s real-time inventory management and accurate demand forecasting [8]. The competence in electronic recording and reporting among healthcare workers impacts medicine supply chain performance by reducing errors and task burden, saving time, influencing the availability of drugs, and improving reporting rates [17,18]. A previous study revealed that the e-LMIS contributes to data accessibility at 94.9% and a reduction in quantification challenges at 57.6%, improves the quality of data by 77.9%, and maintains inventory management of health products at optimum levels, with a decrease in expiry-related losses [19]. In public health facilities, the demand for medications can be highly unpredictable due to factors such as disease outbreaks or changing population health trends. The pharmaceutical supply chain IT system needs sophisticated forecasting and inventory management capabilities to handle these fluctuations. It must be tailored to address logistical issues, ensuring the timely and equitable distribution of medicines.

A prior study reported that the e-LMIS provided real-time health commodity data from user units. The data were essential for medicine supply chain performance as they are used for the planning and management of health commodities, informing the quantification, forecasting, and pipeline monitoring of commodities at all levels of care and decision-making processes [20]. In this study, competence in utilizing the e-LMIS emerges as a critical factor in preventing stockouts and influencing decision-making processes. Real-time insights provided by the system enable participants to anticipate demand accurately, thereby avoiding situations where essential drugs are unavailable. This not only ensures continuous availability but also optimizes resource allocation, minimizing waste and maximizing efficiency. Two key issues limiting competence in e-LIMS utilization reported in this study were shortages of staff and a lack of customized training for health workers. Prior research has identified several limitations, including complexities within the system, an inadequate workforce, authorities’ and users’ accountability, and an inadequate structure to facilitate the organization and management of the health supply chain system [19,21]. The in-depth interviews with the study participants revealed that regular training programs, capacity-building activities, system automation, and simplification are effective ways to enhance the impact of the e-LMIS on the medicine supply chain.

This study serves as a reference point for future studies to assess e-LMIS progress, barriers, and facilitators in various settings and to identify areas in need of customized strategies to improve the utilization of the system. The strength of this study lies in its mixed methods, using both quantitative and qualitative data. By combining statistical analysis with detailed narrative insights, the study provides a comprehensive understanding of healthcare workers’ competence in the e-LMIS and its implications for the medicine supply chain. However, certain limitations should be taken into account when considering the results reported in this research. The cross-sectional design used provides a snapshot of e-LMIS utilization but does not capture long-term trends or establish causality. Self-reporting bias could potentially affect the accuracy of participants’ responses, particularly in assessing their competence levels. Furthermore, a validity test was not performed for the questionnaire used to assess competence; instead, the validity of the questionnaire was based on expert opinion. The study’s area was one district only, which may restrict the generalizability of the findings to other healthcare contexts within Tanzania. Lastly, this study did not explore variations in competence levels among different types of healthcare providers (e.g., nurses, pharmacists, and administrators). Nonetheless, this study serves as a valuable foundational resource for future research and policy development in healthcare supply chain management in the region, offering insights into how to enhance e-LMIS utilization effectively.

## 5. Conclusions

This study found that an adequate proportion of healthcare providers demonstrated competence in utilizing the e-LMIS. Moreover, the study highlighted the positive impact of the e-LMIS on various aspects of the medicine supply chain, including the timely submission of orders and enhanced inventory management. However, challenges such as staff shortages and inadequate training were identified as barriers to the system’s optimal functioning. Regular training, incentive programs, adequate resource allocation, leadership involvement, and the regular utilization of the e-LMIS are crucial for building and maintaining competence among healthcare providers, thereby optimizing the medicine supply chain and ultimately improving healthcare delivery.

## Figures and Tables

**Table 1:** User perceptions of e-LMIS utilization (N = 106).

Variable/Response	Frequency	Percent
Confidence in fulfilling e-LMIS duties		
Very confident	45	42.5
Confident	43	40.6
Somehow confident	17	16.0
Not confident at all	1	0.9
Access to Technology (Facility has a computer)		
Yes (observed)	106	100.0
No	0	0
System Perceptions		
e-LMIS is user-friendly and easy to use		
Yes	85	80.2
No	21	19.8
Orders Placed per Quarter (3 months)		
1 time	66	62.3
2 times	27	25.5
More than 2 times	13	12.3
Timeliness of R&R Revisions		
Yes	72	67.9
No	34	32.1

**Table 2:** Competence levels of study participants on e-LMIS (N = 106).

		Very Competent	Competent	Somehow Competent	Not Competent
1	Ability to search, find, and retrieve information using e-LMIS	5 (4.7%)	38 (35.8%)	55 (51.9%)	8 (7.6%)
2	Evaluation of information obtained from e-LMIS	2 (1.9%)	40 (37.7%)	62 (58.5%)	2 (1.9%)
3	Sorting and organizing information within e-LMIS	1 (0.9%)	32 (30.2%)	69 (65.1%)	4 (3.8%)
4	Manipulating data and generating reports in e-LMIS	2 (1.9%)	31 (29.3%)	70 (66.0%)	3 (2.8%)
5	Adjusting settings and customizing features in e-LMIS based on the needs	3 (2.8%)	38 (35.8%)	62 (58.5%)	3 (2.8%)
6	Familiarity with data visualization tools and techniques	1 (0.9%)	38 (35.8%)	66 (62.3%)	1 (0.9%)
7	Using digital devices and networks to communicate with others	2 (1.9%)	28 (26.4%)	75 (70.8%)	1(0.9%)
8	Sharing and collaborating on information using e-LMIS	8 (7.6%)	38 (35.8%)	55 (51.9%)	5 (4.7%)
9	Interacting with colleagues and stakeholders through e-LMIS	3 (2.8%)	38 (35.8%)	62 (58.5%)	3 (2.8%)
10	Understanding of data sharing and confidentiality protocols	2 (1.9%)	34 (32.1%)	67 (63.2%)	3 (2.8%)
11	Troubleshooting routine hardware and software problems in e-LMIS	3 (2.8%)	33 (31.1%)	67 (63.2%)	3 (2.8%)
12	Seeking digital solutions when encountering difficulties in e-LMIS	4 (3.8%)	30 (28.3%)	70 (66.0%)	2 (1.9%)
13	Protecting digital devices from cyber or physical attacks	3 (2.8%)	32 (30.2%)	68 (64.2%)	3 (2.8%)
Overall assessment		3 (2.8%)	34 (32.1%)	66 (62.3%)	3 (2.8%)

## Data Availability

The data presented in this study are available upon request from the corresponding author.
